# Estimating range expansion of wildlife in heterogeneous landscapes: A spatially explicit state‐space matrix model coupled with an improved numerical integration technique

**DOI:** 10.1002/ece3.4739

**Published:** 2018-12-15

**Authors:** Yutaka Osada, Takeo Kuriyama, Masahiko Asada, Hiroyuki Yokomizo, Tadashi Miyashita

**Affiliations:** ^1^ Graduate School of Life Sciences Tohoku University Sendai Miyagi Japan; ^2^ Graduate School of Agriculture and Life Sciences The University of Tokyo Tokyo Japan; ^3^ Wildlife Management Research Center Hyogo Japan; ^4^ Institute of Natural and Environmental Sciences University of Hyogo Hyogo Japan; ^5^ ASADA Wildlife Management Company (AMAC, LLC) Chiba Japan; ^6^ National Institute for Environmental Studies Ibaraki Japan

**Keywords:** advection, *Cervusnippon*, dispersal‐related demographic stochasticity, Markov chain Monte Carlo with particle filter, spatio‐temporal population dynamics

## Abstract

Dispersal as well as population growth is a key demographic process that determines population dynamics. However, determining the effects of environmental covariates on dispersal from spatial‐temporal abundance proxy data is challenging owing to the complexity of model specification for directional dispersal permeability and the extremely high computational loads for numerical integration. In this paper, we present a case study estimating how environmental covariates affect the dispersal of Japanese sika deer by developing a spatially explicit state‐space matrix model coupled with an improved numerical integration technique (Markov chain Monte Carlo with particle filters). In particular, we explored the environmental drivers of inhomogeneous range expansion, characteristic of animals with short dispersal. Our model framework successfully reproduced the complex population dynamics of sika deer, including rapid changes in densely populated areas and distribution fronts within a decade. Furthermore, our results revealed that the inhomogeneous range expansion of sika deer seemed to be primarily caused by the dispersal process (i.e., movement barriers in fragmented forests) rather than population growth. Our state‐space matrix model enables the inference of population dynamics for a broad range of organisms, even those with low dispersal ability, in heterogeneous landscapes, and could address many pressing issues in conservation biology and ecosystem management.

## 1 INTRODUCTION

For a few decades, state‐space models have provided a powerful methodology for exploring the effects of environmental covariates on population growth and dispersal in wildlife populations from spatio‐temporal count data (Wikle, 2003). These models are widely used in population ecology and conservation biology, such as for evaluating the influence of climate change on species distribution ranges (Pagel & Schurr, 2012), restoring populations of threatened species (Lindley, 2003), establishing effective plans for wildlife management (Iijima, Nagaike, & Honda, 2013; Osada, Kuriyama, Asada, Yokomizo, & Miyashita, 2015), and predicting the range expansion of invasive or pest species (Bled, Royle, & Cam, 2011; Hooten & Wikle, 2008; Veran et al., 2015; Wikle, 2003). By explicitly separating the observation processes from population dynamic processes, state‐space models explore useful information about the underlying non‐stationary dynamics of wildlife (Guisan & Thuiller, 2005; Pagel & Schurr, 2012).

In wildlife populations, dispersal is a key demographic process especially when the target population forms a metapopulation structure or shows range expansion dynamics (Hanski & Thomas, 1994; Hastings et al., 2005). The dispersal process has been explicitly modeled by so‐called matrix models (or first‐order Markov models) in previous state‐space modeling studies (Conn et al., 2015; Hooten, Wikle, Dorazio, & Royle, 2007; Pagel & Schurr, 2012; Wikle, 2003; Williams et al., 2017). In particular, these studies modeled the dispersal as the result of diffusion process to describe expanding and/or shifting habitat range. While the development is critical to non‐stationary spatio‐temporal modeling, there still remain two challenging difficulties for assessing the dispersal process using state‐space models.

The first difficulty is the complexity of model specification for directional dispersal permeability (i.e., advection) originating from spatially variable environments. If environmental elements have negligible effects on advection, the dispersal probability between locations is approximately expressed by symmetric Gaussian or long‐tailed exponential kernels (i.e., isotropic dispersal permeability, or diffusion), which are widely used for animals with relatively high dispersal ability, such as birds and insects (Bled et al., 2011; Prasad et al., 2010; Wikle, 2003). However, such an approximation may be invalid for most mammals and amphibians because their dispersal can be directional depending on environmental elements (Coulon et al., 2006; Niedziałkowska, Fontaine, & Jędrzejewska, 2012; Pérez‐Espona et al., 2008). In this situation, we need to specify the probability of dispersal between locations considering the presence of an infinite number of dispersal pathways (Ovaskainen, 2008). To illustrate this issue, we consider a simple case of animal dispersal from location A to B. If the animal disperses directly between habitats, the pathway is simply A → B. However, when the animal passes through another location C, the process becomes much more complicated because the potential pathways increase exponentially (for example, A → C → B, A → C → A → B, and so on). Furthermore, the permeability of these pathways may be different (e.g., between A → B and A → C) and the dispersal direction may be asymmetric (e.g., between A → C and C → A) owing to environmental heterogeneity in the landscape. The second difficulty with incorporating dispersal process into state‐space models is the extremely high computational load for the numerical integration of posterior densities (or likelihoods) when modeling demographic stochasticity in the dispersal process. Imagine the two‐habitat situation, in which 95% of individuals stay in their native habitat and 5% disperse to the other habitat. If there are 10 individuals before dispersal, the number of dispersed individuals will be around the expectation of 0.5 according to binomial distribution, Bin (10, 0.05). We refer to this variation as dispersal‐related demographic stochasticity. In many‐habitat situations, this dispersal‐related demographic stochasticity can be addressed by multinomial distribution (i.e., the extension of binomial distribution). Using common techniques, including the Markov chain Monte Carlo (MCMC) method, it is usually virtually impossible to obtain the posterior probability of population growth and dispersal parameters under dispersal‐related demographic stochasticity. Recently, a numerical technique combining MCMC with particle filters (PFMCMC) has been used effectively to estimate complex population state‐space models (Knape & de Valpine, 2012). However, it is unclear whether PFMCMC is also effective for state‐space modelling with dispersal because increasing the number of locations (or state variables) may lead to a notorious problem known as “particle shrinkage” in the particle filter algorithm (See Supporting Information Appendix C for details).

In this paper, we present a case study estimating how environmental covariates affect the dispersal of a wildlife pest, Japanese sika deer (*Cervus nippon*). We circumvent the above modeling issues by developing a state‐space matrix model and an improved method for the numerical integration of particle filters. Japanese sika deer are undergoing range expansion, and this is a major target for wildlife management in most parts of Japan. Their browsing and grazing behaviors are known to clear forest‐floor vegetation, harm the bark of timber trees, and cause agricultural crop damage (Agetsuma, 2007). We are particularly interested in the drivers of the inhomogeneous range expansion of sika deer under a heterogeneous landscape, which may be explained by differences in population growth among habitats, differences in permeability among dispersal pathways or both. The main aim of our model was to specify movement on an extremely short, unobservable timescale; annual dispersal is expressed as repeated short‐term movements, in which we can assume that animals either stay at the same location or move to neighboring locations. Our model development allows us to reproduce the circumventions of dispersal barriers in range expansion dynamics by modeling directional dispersal permeability.

In our case study, we used long‐term monitoring data for Japanese sika deer in central Japan obtained in 2000–2010. Based on previous findings, our model accounted for the effects of broad‐leaf forest area and forest edge length on deer population growth (Conradt, Clutton‐Brock, & Guinness, 1999; Iijima et al., 2013; Miyashita et al., 2008; Weerasinghe & Takatsuki, 1999) as well as the effects of the total forest area (i.e., both broad‐leaf forest and coniferous plantation area) and the presence/absence of rivers on deer dispersal (Coulon et al., 2006; Niedziałkowska et al., 2012; Pérez‐Espona et al., 2008). To the best of our knowledge, this is the first study to explicitly incorporate the effects of environmental covariates on advection into hierarchical state‐space models of wildlife population dynamics.

## 2 METHODS

### 2.1 Study site

**Figure 1:**
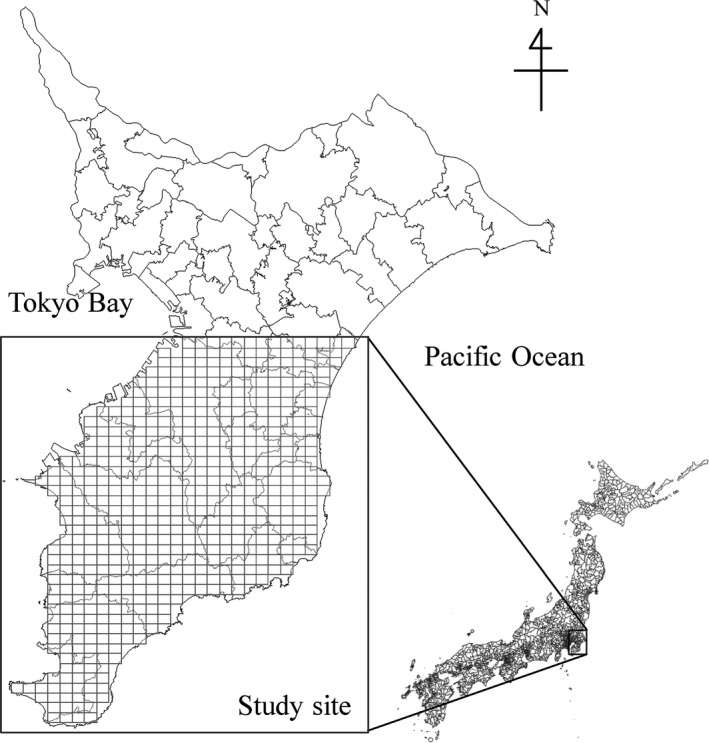
Study area in Boso peninsula, central Japan. The gray grid cells represent analytical units (2 × 2 km^2^) in which we estimated the population dynamics of sika deer. Boso peninsula is surrounded by Tokyo Bay and the Pacific Ocean

Our study site was located in Boso peninsula, Chiba, central Japan (34°91′–35°55′N, 139°75′–140°47′E; Figure 1). The climate is warm temperate, with a monthly mean temperature of 4–25°C and a mean annual precipitation of 1,400 mm. Snow accumulation affects sika deer mortality and dispersal (Igota et al., 2004; Jędrzejewski, Jędrzejewska, Okarma, & Ruprecht, 1992), but deep snow is uncommon in this area. The dominant vegetation is characterized by a broad‐leaf evergreen forest of *Castanopsis sieboldii* and coniferous plantations of *Cryptomeria japonica*. There are no major predators of sika deer, such as wolves, in the Boso peninsula. The sika deer population in this area was once isolated to a restricted area by pressure from urban development and overhunting, but the population began increasing in the 1970s to 1980s and the range has since expanded (Asada, 2014). The Chiba prefectural government administered an implementation plan to control sika deer abundance with monitoring since 1996 (Chiba Prefectural Government, 2015).

From published reports and unpublished records of the prefectural government, we compiled sika deer abundance proxies (fecal pallet count survey in 2000–2010, Supporting Information Figure S1; block count survey in 2000–2008, Supporting Information Figure S2a–i; and preferred plant damage survey in 2001, Supporting Information Figure S2 *j*) and the number of hunted sika deer in 2000–2009 (Supporting Information Figure S3). As environmental factors affecting deer population growth and dispersal process, broad‐leaf forest area, forest edge length, total forest area (both broad‐leaf forest and coniferous plantation area), and the presence/absence of rivers were extracted using a geographical information systems approach (Supporting Information Figure S4). The details of data collection and preliminary processing are described in Appendix A.

## 3 MODEL FORMULATION

**Figure 2:**
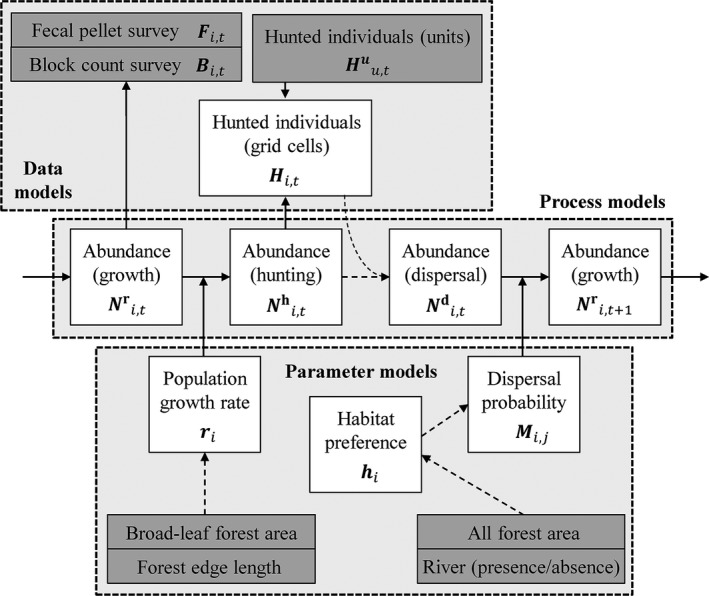
Schematic diagram of the model structure. White and gray boxes represent estimated quantities and data, respectively. Our model consists of three component models: process models, parameter models, and data models. Solid and dashed arrows represent stochastic and deterministic relationship between boxes

A state‐space matrix model was developed to describe spatio‐temporal changes in sika deer abundance at 578 discretized grid cells (ca. 2 × 2 km^2^; Figure 1) in 2000–2010. Our state‐space matrix model is explained by three component models: process models, parameter models, and data models (Berliner, 1996; Pagel & Schurr, 2012; Wikle, 2003; Figure 2). In this section, the vector notation ***x*** *_i_*_∈_ *_S_* = {*x_i_|i*∈*S*} is used (*S* is the set of vector elements). The grid size was determined from the daily deer movement capacity (Chiba Prefectural Government, 2004).

### 3.1 Process models (population dynamics model)

Our process models describe the population dynamics of sika deer considering demographic stochasticity. In the study area, sika deer dynamics are influenced by three main demographic processes: population growth (birth and natural mortality in spring and summer), hunting mortality (in fall and winter), and dispersal (in winter). Let $N_{i,t}^{r}$, $N_{i,t}^{h}$, and $N_{i,t}^{d}$ be the deer abundance before population growth, hunting mortality, and dispersal process at grid cell *i* in year *t*. Poisson and multinomial distributions were used to model demographic stochasticity related to population growth and dispersal, respectively. Using the population growth rate at grid cell *i* (*r_i_*) and dispersal probability from grid cell *i* to *j* (*M_ij_*), process models were specified as follows:

(i) population growth process:

$$N_{i,t}^{h}∼\text{Pois}\;\left(r_{i}N_{i,t}^{r}\right);$$

(ii) hunting mortality process:

$$N_{i,t}^{d}=N_{i,t}^{h}-H_{i,t},$$

where *H_i,t_* is the number of hunted individuals at grid cell *i* in year *t*; and

(iii) dispersal process:

$$N_{i,j\in \{1,2,\ldots ,578\},t}^{m}∼\text{Multin}\;\left(N_{i,t}^{d};M_{i,j\in \{1,2,\ldots ,578\}}\right),$$

$$N_{i,t+1}^{r}=\sum_{j=1}^{578}N_{j,i,t}^{m},$$

where $N_{j,i,t}^{m}$ is the number of dispersed individuals from grid cell *j* to *i* in year *t*. We incorporated dispersal‐related demographic stochasticity (represented by the multinomial distribution) into the process model because range expansion to new locations is strongly determined by chance, particularly for relatively small grid sizes. Note that $N_{i,t}^{h}\geq H_{i,t}$ is necessarily satisfied at each grid.

The deer distribution range in 2000 is considered to reflect earlier historical range expansion (Osada et al., 2015; Williams et al., 2017). Thus, we modeled spatial autocorrelation in the initial distribution range using a Poisson distribution as follows:

$$N_{i,2000}^{r}∼\text{Pois}\left(A_{i}\frac{\sum_{j\in n_{i}}N_{j,2000}^{r}}{\sum_{j\in n_{i}}A_{j}}\right)$$

where *A_i_* is the deer habitat (i.e., broad‐leaf forest and coniferous plantation) area at grid cell *i* and *n_i_* is the set of adjacent grid cells to grid cell *i*. Because damage of the preferred plant *Aucuba japonica* in 2001 is a reliable proxy of sika deer presence/absence (Suzuki, Miyashita, Kabaya, Ochiai, & Asada, 2008; Appendix A), we set $N_{i,2000}^{r}$ to zero if no feeding damage was observed at grid cell *i*.

### 3.2 Parameter models

Our parameter models describe the relations between sika deer demographic parameters (population growth rate and dispersal probability) and environmental covariates. First, we expressed population growth rates by standardized broad‐leaf forest area (BLF*_i_*) and standardized forest edge length (EDGE*_i_*):

$$\log\;\left(r_{i}\right)=\alpha_{0}+\alpha_{1}{\text{BLF}}_{i}+\alpha_{2}{\text{EDGE}}_{i},$$

where ***α*** = {*α*_0_, *α*_1,_ *α*_2_} is the intercept and coefficients of environmental covariates. Because a sika deer female produces one calf each year at most, we imposed an additional constraint on ***α*** such that *r_i_* must be <1.5

Second, we expressed the dispersal probability (*M_i,j_*) by standardized total forest area and presence/absence of rivers. For this purpose, we define $M_{i,j}^{\Delta }$ as the short‐term movement probability during a fraction of a year, ∆*t*. Considering the short‐term movement probability (with a sufficiently short ∆*t*), we can ignore the dispersal between distant grid cells, and readily specify this probability by the dispersal distance between adjacent grid cells (DIST*_i,j_*) and environmental covariates (standardized total forest area, FRT*_i_*, and presence/absence of rivers, RIV*_i_*):

$$h_{\mathrm{ij}}=\beta_{0}\;{\text{DIST}}_{i,j}+\beta_{1}{\text{FRT}}_{j}+\beta_{2}{\text{RIV}}_{j},$$

$$M_{i,j}^{\Delta }=\frac{e^{h_{i,j}}}{\sum_{k\in \{i,n_{i}\}}e^{h_{k,j}}},$$

where ***β*** = {*β*_0_, *β*_1_, *β*_2_} denotes the coefficients of dispersal distance and environmental covariates. If habitat preference, *h_i,i_*, is positive, sika deer stay in grid cell *i* more frequently than by chance. On the other hand, negative habitat preference means that sika deer move from grid cell *i* more frequently than by chance. Owing to the distance decay of dispersal rates, we assumed that *β*_0_ must be negative in our model. The annual dispersal probability can be expressed as repeated short‐term movement:

$$M={\left(M^{\Delta }\right)}^{1/\Delta t}$$

We used ∆*t* = 1/16 year (i.e., 22.8 days) in our analysis, which appeared to be a sufficiently short time period compared to the observed speed of sika deer range expansion. As most elements of the short‐term movement probability are zero, the annual dispersal probability can be efficiently calculated by sparse matrix implementation. We chose a reflective boundary condition for considering dispersal probability across the edge of our study area (Conn et al., 2015; Williams et al., 2017). This choice was trivial in our results because sika deer abundance was zero at the boundary during our study periods. Our modeling of dispersal probability naturally specifies advection and diffusion by using a parameter model, although we can also express these dispersal processes separately by different parameter models.

### 3.3 Data models

Our data models describe the relations between sika deer abundance before population growth ($N_{i,t}^{t}$) and its proxies from the fecal pellet count survey (*F_i,t_*) and block count survey (*B_i,t_*). We modeled negative binomial or Poisson distributions as the detection uncertainty related to each survey. Using a proportionality constant between sika deer densities and fecal pellet counts (*γ* > 0) and the dispersion parameter of fecal pellet counts (*θ* > 0), data models of abundance proxies were obtained as follows:

$$F_{i,t}∼\text{Negbin}\left(\theta ,\frac{\theta A_{i}}{\gamma N_{i,t}^{r}}\right)\left(i\in f_{t}\right),$$

$$B_{i,t}∼\text{Pois}\left(A_{i}^{B}\frac{N_{i,t}^{r}}{A_{i}}\right)\left(i\in b_{t}\right),$$

where $A_{i}^{B}$ is the unit area of block count survey (0.95–2.21 km^2^) and *f_t_* and *b_t_* are the sets of grid cells in which a fecal pellet count survey and block count survey were conducted in year *t*, respectively. Note that our notation for the negative binomial distribution uses shape and inverse‐scale parameters. The overdispersion of fecal pellet counts is considered to result from local microtopography and microclimate.

Records of hunted sika deer were summarized for 66 management units (4.22–109.92 km^2^) by government officials (see details in Appendix A). Thus, we additionally modeled the data uncertainty related to the number of hunted individuals at each grid cell (*H_i,t_*). Assuming that the hunting rate was constant at each grid cell within a management unit (i.e., hunted numbers are proportional to abundance at grid cells), we expressed this data uncertainty with a multinomial distribution as follows:

$$p_{i,t}=\frac{N_{i,t}^{h}}{\sum_{i\in m_{k}}N_{i,t}^{h}}\left(i\in m_{k}\right),$$

$$H_{i\in m_{k},t}∼\text{Multin}\;\left(H_{k,t}^{u};p_{i\in m_{k},t}\right),$$

where $H_{k,t}^{u}$ is the number of hunted individuals at management unit *k* in year *t* and *m_k_* is the set of grid cells included in management unit *k*. Based on our assumption, different hunting rates are assured among different management units.

### 3.4 Model assimilation

The full specification of posterior and prior probabilities is described in Appendix B. We set uniform or vague normal distributions as the prior probabilities of estimates (Supporting Information Table S1) and checked that all prior distributions were weakly informative by result summaries and trace plots (Supporting Information Figure S5). Owing to the high computational cost of modeling multiple dispersal pathways and dispersal‐related demographic stochasticity in the integration of the posterior probability, we implemented Bayesian inference via the PFMCMC algorithm (Andrieu, Doucet, & Holenstein, 2010; Knape & de Valpine, 2012). Our PFMCMC algorithm is largely consistent with that of Knape & de Valpine (2012), but the particle filter algorithm was modified to approximate the appropriate posterior probability (see details of the PFMCMC algorithm and our modification in Appendix C). Our modification dramatically improved particle approximation by the direct sampling of state variables in the first year from the filtered probability (Supporting Information Figure S6–7).

We ran eight MCMC chains for 10,000 iterations after finishing the adaptive stage according to Vrugt et al. (2009). To obtain the summaries of posterior distributions, we sampled the MCMC simulations once every 50 iterations and defined credible intervals (CIs) as the highest posterior density intervals. We confirmed Gelman–Rubin convergence diagnostics $\hat{R}$ <1.1 for all parameters. The goodness‐of‐fit of our model was assessed based on posterior predictive *p* value (or Bayesian *p* value), measured by Freeman‐Tukey statistic (Kéry & Royle, 2016).

## 4 RESULTS

**Figure 3:**
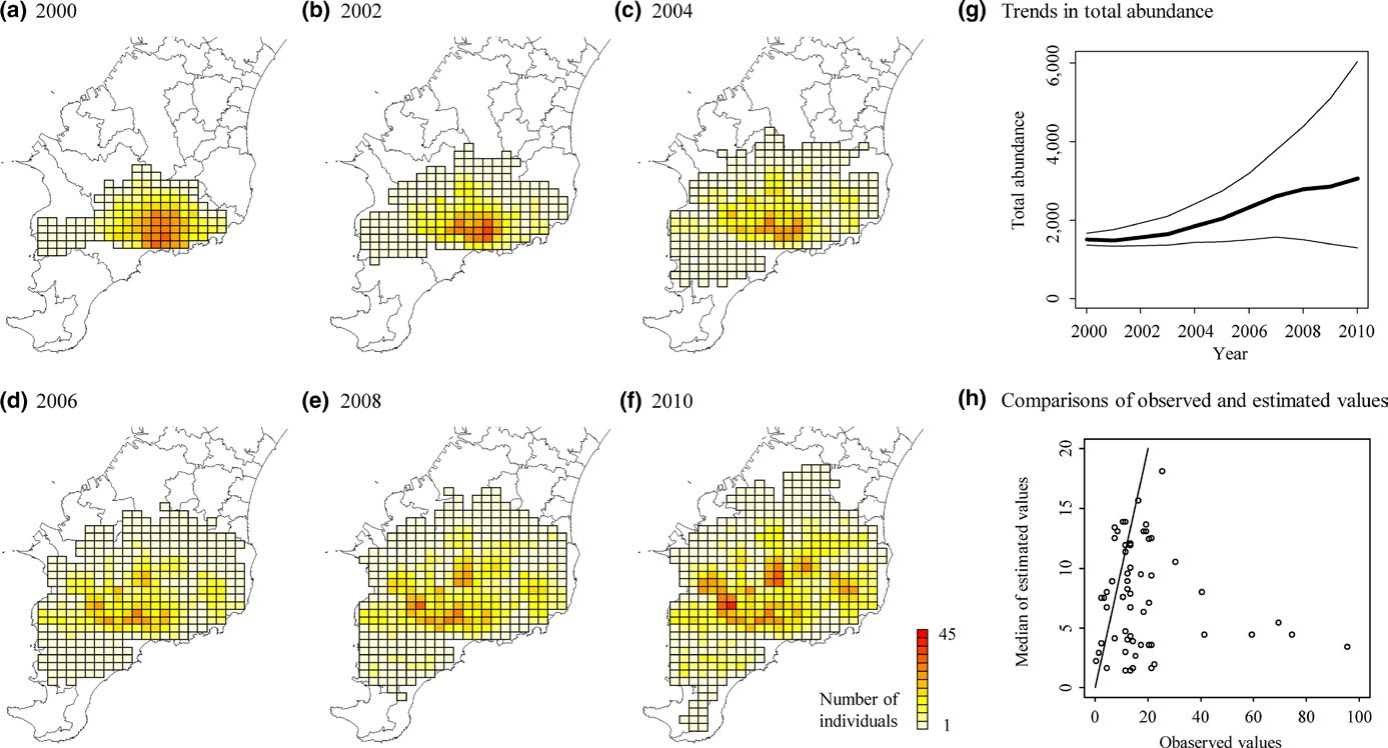
Estimated trends in sika deer abundance at analytical grid cells. Our results showed that sika deer expanded their distribution range (a–f) and increased in total abundance (g) in 2000–2010. In panel g, bold and thin lines represent the median and 50% credible interval of the estimated total abundances, respectively. We used the 50% credible interval to clarify trends in total abundances. To check model fitting, we conducted comparisons between observed and estimated abundances obtained from block count surveys in 2000–2008 (h). A solid line represents identity line. Although we failed to fit a survey unit (of different years) with extraordinarily high density (i.e., 40–95 observed values) among fifteen survey units, most estimates were well fitted to observed values

Our results showed that sika deer expanded their distribution range and increased from 1,506 individuals in 2000 to 3,054 in 2010 in the southern Boso peninsula (Figure 3). Sika deer clearly exhibited inhomogeneous range expansion (Figures 3a–f). The 50% CIs of total abundance in 2000–2002 was narrow but those in 2010 were comparatively wide (Figure 3g). The goodness‐of‐fit estimated by posterior predictive *p* was 0.306, suggesting no lack of model fit. We also checked our model fitting by the comparison between observed abundance from block count survey and our estimates (Figure 3h). Most estimates were well fitted to the observed data, but the fitting was not greatly successful, as one of the survey units (of different years) with extraordinarily high densities exhibited profound difference between data and estimates. For the fecal pellet count survey, 36.4 fecal pellets correspond to an individual sika deer on average, and the dispersion parameter (i.e., detection uncertainty) was 0.368 (Table 1).

**Table 1:** Summary of our state‐space model: descriptions and posterior medians (95% credible intervals) of model parameters

	Description	Posterior median [95% credible interval]
*γ*	Proportionality constant used for conversion between sika deer densities and fecal pellet counts	36.38 [6.43, 90.33]
*θ*	Overdispersion parameter for fecal pellet counts	0.368 [0.083, 1.590]
*α*_0_	Intercept of population growth function	0.332 [0.206, 0.390]
*α*_1_	Effect of broad‐leaf forest area on population growth	0.008 [−0.018, 0.055]
*α*_2_	Effect of forest edge length on population growth	−0.004 [−0.058, 0.022]
β_0_	Effect of distance on movement	−1.852 [−2.120, −0.102]
β_1_	Effect of forest area on movement	0.247 [−0.315, 1.170]
β_2_	Effect of rivers on movement	−0.184 [−1.006, 0.709]

**Figure 4:**
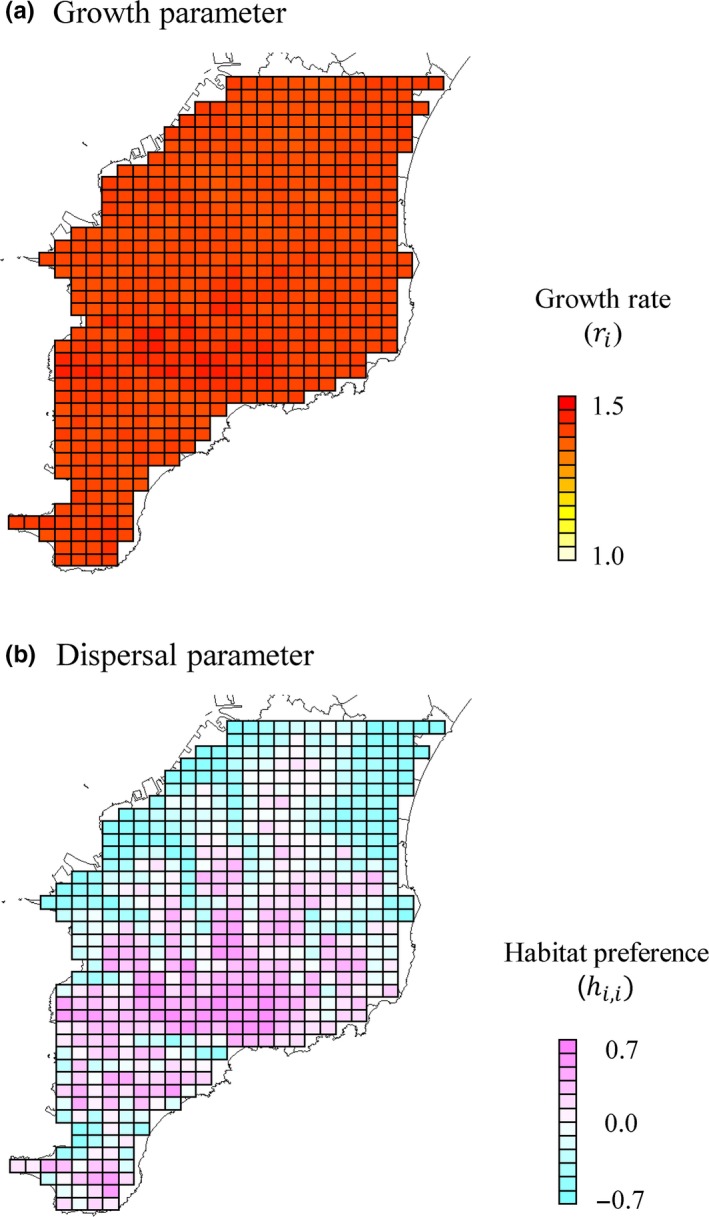
Maps of the estimated population growth rate (a) and habitat preference with respect to movement (b). White to red colors represent low to a high population growth rate, while blue to red colors represent a negative to positive dispersal habitat preference

We found no significant effects of environmental covariates on demographic parameters of sika deer, whose 95% CIs overlapped with zero (Table 1); the effects of broad‐leaf forest area and forest edge length on deer population growth rate were 0.008 and −0.004, and the effects of total forest area and the presence/absence of rivers on the dispersal habitat preference were 0.247 and −0.18. We detected an inhomogeneous spatial structure of dispersal habitat preference (Figure 4b), but population growth rates were constant in most of our study area (*r_i_* ≈ 1.39; Figure 4a). The distance effect on movement preference was estimated to be −1.852, indicating that mean dispersal distance of sika deer is about 6.5 km per year if the landscape was homogeneous.

## 5 DISCUSSION

By expanding a previous state‐space matrix model to address the effect of environmental covariates on directional dispersal permeability and dispersal‐related demographic stochasticity, we investigated how different environmental elements affect not only population growth but also dispersal of Japanese sika deer in a heterogeneous landscape. We used species‐specific assumptions in our model to prevent biologically unrealistic estimates (e.g., sika deer cannot bear two or more calves in a year). However, these assumptions can be readily modified and extended to fit available data and characteristics of focal species (e.g., Hostetler & Chandler, 2015). Because our model can estimate population dynamics even for organisms with low dispersal ability in heterogeneous landscapes, our model has a wilder applicability to many organisms in the wild.

The most important finding of our results is that forest area seemed to have a positive effect on dispersal and lead to an inhomogeneous spatial structure (Figure 4b), suggesting that small, fragmented forests may act as movement barriers. Although the significance level was marginal (0.090), the effect size was relatively large. This finding agrees with evidence from previous deer studies (Niedziałkowska et al., 2012; Sakuragi et al., 2003). Niedziałkowska et al. (2012) indicated that natural or semi‐natural forest habitats provide nesting vegetation and resources to sika deer, but forest habitats with high hunting pressure and man‐made construction induce strong avoidance behaviors (Takii, Izumiyama, Mochizuki, Okumura, & Sato, 2012). Because our study area is characterized by relatively low human activity, sika deer may gain behavioral advantages from forest habitats.

We also found that sika deer exhibited high, homogeneous population growth in their distribution range. Although broad‐leaf forests and forest edges are known to provide abundant food resources, such as nuts and edge vegetation, in our study area (Asada & Ochiai, 1996; Miyashita et al., 2008) and in other study areas (Weerasinghe & Takatsuki, 1999), we did not detect effects of these habitat types at the population level. As the estimated population growth rate was around 1.39, which is close to the maximum (1.5), the lack of effects of environmental factors on the population growth rate is probably explained by the sufficient food supply throughout the study area. Another possibility of homogeneous population growth is that we could not detect significant effects due to low estimability arising from model complexity and limited available data. As explained below, high overdispersion and spatial sparseness of our data make it difficult to simultaneously estimate population growth and dispersal process. However, given the striking spatial structure of dispersal process (Table 1, Figure 4b), our results supported the conclusion that the inhomogeneous range expansion of sika deer in the Boso peninsula mainly resulted from the dispersal process, rather than the population growth process.

The estimated total population size of sika deer displayed high uncertainty (Figure 3). This uncertainty appeared to reflect the high overdispersion and spatial sparseness of abundance proxy data. In our negative binomial model, the estimated variance of fecal pellet counts was about 100 × (population density) times larger than that of the Poisson model. This result suggests that our fecal pellet count survey may provide a rough estimate of population density in areas in which deer are abundant. The data dispersion could be improved by capturing influential local environmental covariates and/or modifying survey protocols. The data sparseness led to many unobserved habitat areas in space‐state matrix models that include many small grid cells. In our case study, grid cells with fecal pellet counts accounted for 14.3% of the whole area (910 counts/578 grid cells/11 years). The population density in the unobserved area thus exhibited high estimation errors, resulting in a high uncertainty for total population size and demographic parameters (i.e., the effects of environmental factors on population growth and dispersal). It is important to point out that the success of previous state‐space matrix models (e.g., Hooten et al., 2007; Pagel & Schurr, 2012; Conn et al., 2015; Williams et al., 2017) may be partially attributed to spatially intensive observations. When using sparse spatio‐temporal count data, we will need careful specification of informative priors for abundance by conducting preliminary surveys or collecting data from other sources.

For dispersal process, advection and diffusion are definitely distinguished by directionality of movement. An earlier study developed model framework with variable diffusion rate and accounted for colonization dynamics of sea otters (Williams et al., 2017). To our knowledge, this paper is the first study to explicitly account for the effects of environmental covariates on the directional dispersal permeability (i.e., advection) in a state‐space model of wildlife population dynamics. Because our modeling approach pose the challenging numerical computation of posterior probabilities, computational improvement is essential for estimating spatio‐temporal wildlife dynamics that are affected by environmental drivers at various spatial and temporal scales. The PFMCMC algorithm is a promising tool for such complex state‐space models, but it has only been applied to a few population models to date (Knape & de Valpine, 2012). Our modification of a numerical integration technique for particle filtering allows us to use the PFMCMC algorithm more effectively for applications to ecological issues. We provide theoretical validation and simple numerical experiments in Appendix C; further studies are needed to evaluate its application to more complex numerical experiments with realistic conditions.

Despite the urgent demand for the conservation and management of mammalian and amphibian taxa, few studies have estimated the effects of environmental covariates on directional dispersal permeability at an ecological timescale. An important future development of state‐space model is to address density‐dependent dispersal. This is still computationally impractical in our modeling, but applying rough approximation (e.g., ignoring dispersal‐related demographic stochasticity; Wikle, 2003; Williams et al., 2017) may help in modeling density‐dependent dispersal. In a recently changing world, our modeling framework may become increasingly important because many animals, including native and non‐native species, move or expand their distribution ranges by global warming and land‐use changes. Our modeling framework can be applied to address many pressing issues in population ecology and conservation biology.

## CONFLICT OF INTEREST

None declared.

## AUTHOR CONTRIBUTIONS

YO conceived the study, organized field data, carried out the statistical analyses and drifted the manuscript. TK collected and organized field data. MA coordinated the study and collected field data. HY participated in the design of the study and helped draft the manuscript. TM designed the study, coordinated the study and drifted the manuscript. All authors gave final approval for publication.

## DATA ACCESSIBILITY

All the data are available at Dryad digital repository (https://doi.org/10.5061/dryad.pt38f8s).
