# Supplementary material for: Estimating range expansion of wildlife in heterogeneous landscapes: A spatially explicit state‐space matrix model coupled with an improved numerical integration technique
Source: Ecol Evol. 2018 Dec 15;9(1):318–27. doi: 10.1002/ece3.4739 (PMC6342096; doi:10.1002/ece3.4739)
Supplement: Supplementary file 1 [file ECE3-9-318-s001.docx]

**Electronic Supplementary Materials**

Estimating the environmental effects on inhomogeneous range expansion of Japanese sika deer from spatial-temporal data.

Y. Osada, T. Kuriyama, M. Asada, H. Yokomizo and T. Miyashita

**Appendix A: Detailed descriptions of data collection and preliminary processing**

**A-1. Proxies of deer abundance**

We collected sika deer abundance proxies from the previous surveys conducted in 2000-2010 (fecal pellet count surveys and block count surveys; Chiba Prefectural Government 2004). The fecal pellet count surveys have been conducted over 50 survey routes (each 1 km in length) in December and January each year since 1996 (Fig. S1 *a*-*k*). Because of limited budget, the surveys were planned to cover the entire sika deer distribution within 2–3 years. In concert with the observed deer range expansion, the number of established survey routes increased from 119 in 2000 to 167 in 2010. The fecal pellet survey data contained an outlier, determined by local outlier factor, which we excluded from our statistical analysis. The block count surveys were conducted in 15 survey units (0.95–2.21 km^2^) during winter each year in 1996–2008 (Fig. S2 *a*-*i*). Each survey unit was divided into about 20 blocks, and observers recorded the number of sika deer detected in each block within 90 min, while keeping in contact with the other observers to prevent double counting.

To improve the estimation in the deer abundance in 2000, we used data from leaf damage surveys of the preferred forage plant (*Aucuba japonica*), which occurred throughout the entire deer distribution range in 2001 (Chiba Prefectural Government 2004; Fig. S2 *j*). *A. japonica* is a very sensitive plant species, and can be used as a good indicator for the presence of deer at a local scale (Suzuki et al. 2008).

**A-2. Numbers of hunted sika deer**

The numbers of hunted sika deer in 2000–2009 were collected from unpublished governmental records (Fig. S3 *a*-*j*). The preauthorized hunters shoot sika deer only during hunting seasons and reported the hunted numbers to local governmental offices. These records were summarized for 66 management units by government officials. Most of management units (4.22–109.92 km^2^) were established by splitting municipalities in Chiba prefecture. Note that the management units are different from the block count survey units.

**A-3. Environmental factors**

Using geographical information systems, we extracted environmental factors that might affect sika deer population growth and dispersal processes (Fig. S4 *a*-*d*). Total forest area (i.e., both broad-leaf and conifer forest area), broad-leaf forest area, and forest edge length per 1 km^2^ were obtained from the 6th and 7th National Survey on the Natural Environment (available online from http://www.biodic.go.jp/kiso/fnd_f.html), and the presence/absence of rivers was obtained from the Fundamental Geospatial Data of Japan (available online from http://www.gsi.go.jp). We standardized all environmental factors except for the presence/absence of rivers (i.e., scaled and shifted to mean 0 and variance 1). We expected that broad-leaf forest area and forest edge length increase deer population growth (Conradt et al. 1999; Weerasinghe & Takatsuki 1999; Miyashita et al. 2008; Iijima et al. 2013), and total forest area and presence/absence of rivers affect their movements (Coulon et al. 2006; Perez-Espona et al. 2008; Niedziałkowska et al. 2012).

**References**

Chiba Prefectural Government. 2004 *Scientific report on the management of Japanese sika deer in Chiba prefecture* (in Japanese). Chiba, Japan: Chiba Prefectural Government Office.

Conradt L, Clutton-Brock TH, Guinness FE. 1999 The relationship between habitat choice and lifetime reproductive success in female red deer. *Oecologia* **120**, 218-224.

Coulon A, Guillot G, Cosson JF, Angibault JMA, Aulagnier S, Cargnelutti B, Galan M, Hewison AJM. 2006 Genetic structure is influenced by landscape features: empirical evidence from a roe deer population. *Mol. Ecol.* **15**, 1669-1679.

Iijima H, Nagaike T, Honda T. 2013 Estimation of deer population dynamics using a Bayesian state-space model with multiple abundance indices. *J. Wildl. Manage.* **77**, 1038-1047.

Miyashita T, Suzuki M, Ando D, Fujita G, Ochiai K, Asada M. 2008 Forest edge creates small-scale variation in reproductive rate of sika deer. *Popul. Ecol.* **50**, 111-120.

Niedzialkowska M, Fontaine MC, Jedrzejewska B. 2012 Factors shaping gene flow in red deer (*Cervus elaphus*) in seminatural landscapes of central Europe. *Can. J. Zool.* **90**, 150-162.

Perez-Espona S, Perez-Barberia FJ, McLeod JE, Jiggins CD, Gordon IJ, Pemberton JM. 2008 Landscape features affect gene flow of Scottish Highland red deer (*Cervus elaphus*). *Mol. Ecol.* **17**, 981-996.

Suzuki M, Miyashita T, Kabaya H, Ochiai K, Asada M. 2008 Deer density affects ground-layer vegetation differently in conifer plantations and hardwood forests on the Boso Peninsula, Japan. *Ecol. Res.* **23**, 151-158.

Weerasinghe UR, Takatsuki S. 1999 A record of acorn eating by sika deer in western Japan. *Ecol. Res.* **14**, 205-209.


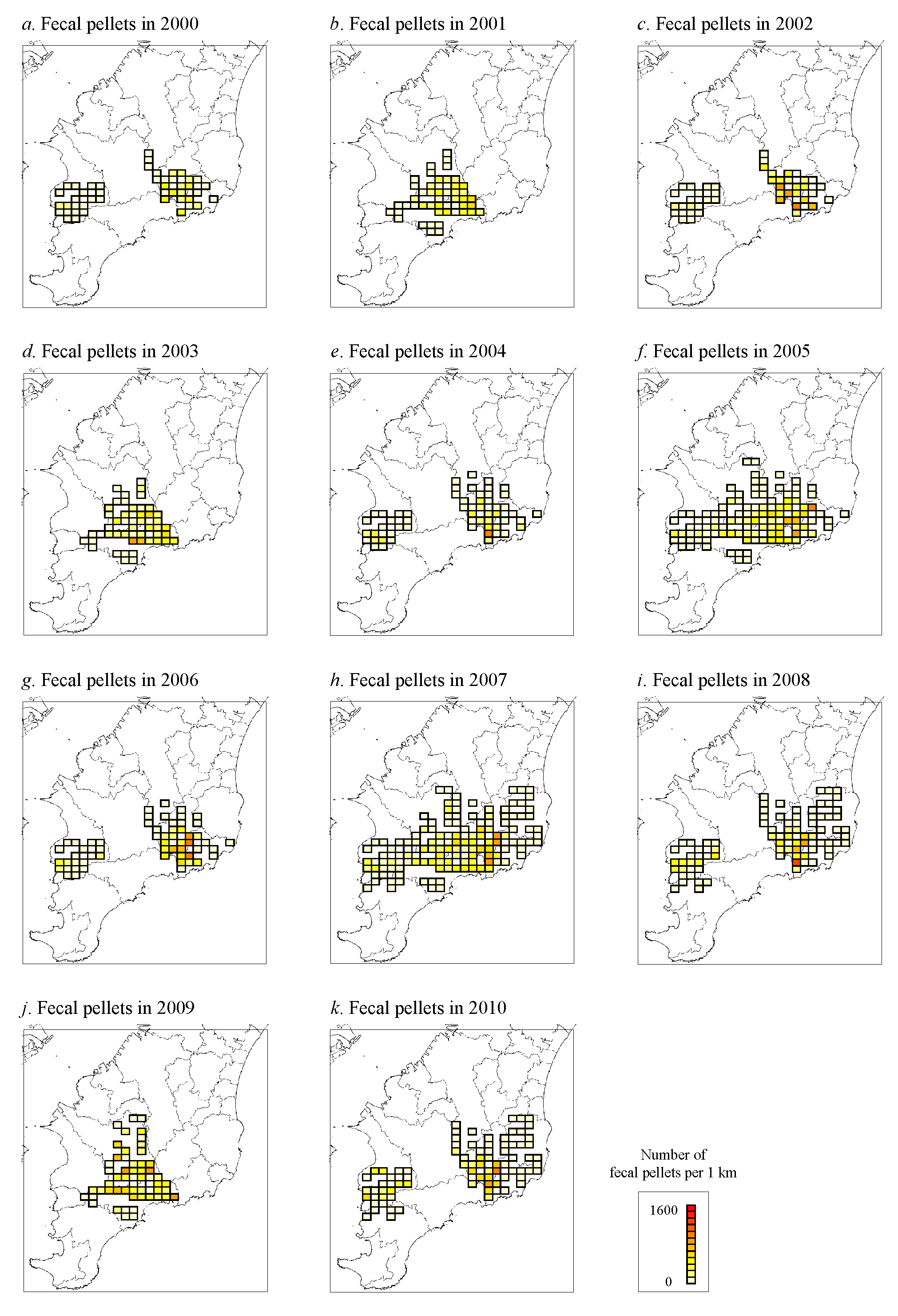


**Figure S1**. The numbers of fecal pellets at 1 km survey routes in 2000-2010.


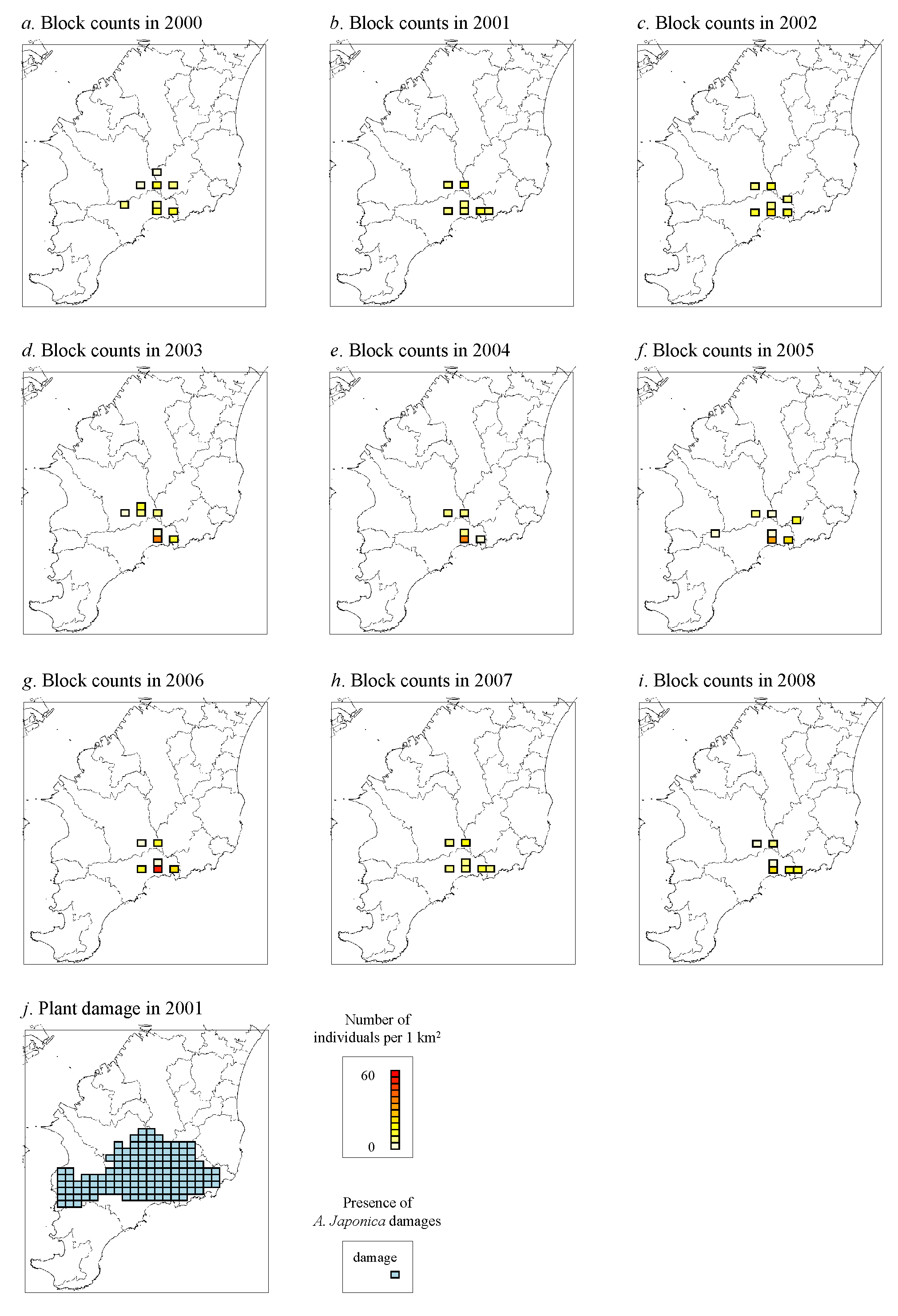


**Figure S2**. The numbers of individuals per 1 km^2^ in 2000-2008 (*a*-*i*) and the damages of preferred plant (*Aucuba japonica*) in 2001 (*j*). The numbers of individuals were counted by block count surveys.


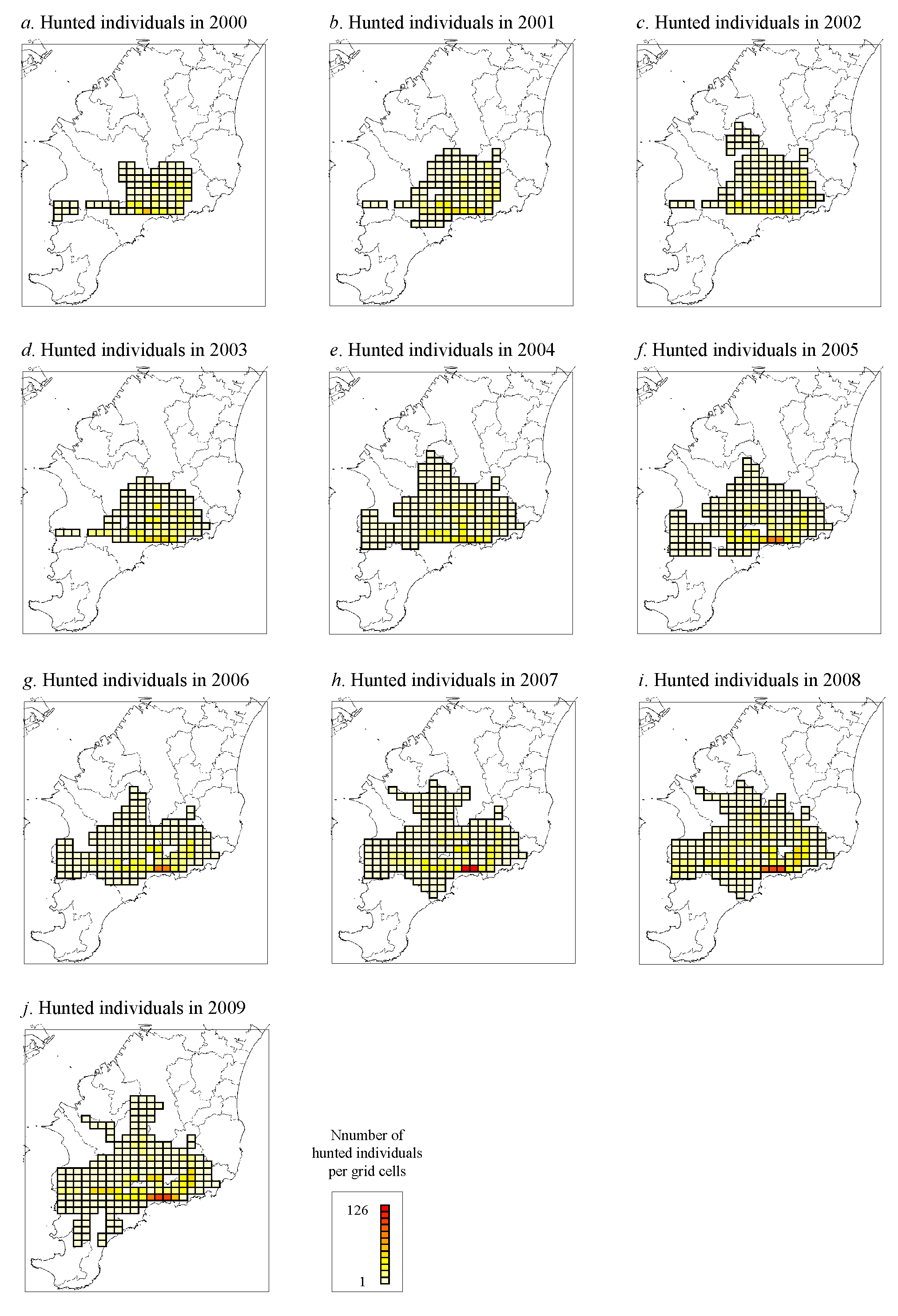


**Figure S3**. The numbers of hunted individuals per grid cells in 2000-2009. To allow readily comparing the other data, the number of hunted individuals each management unit is divided into grid cells.


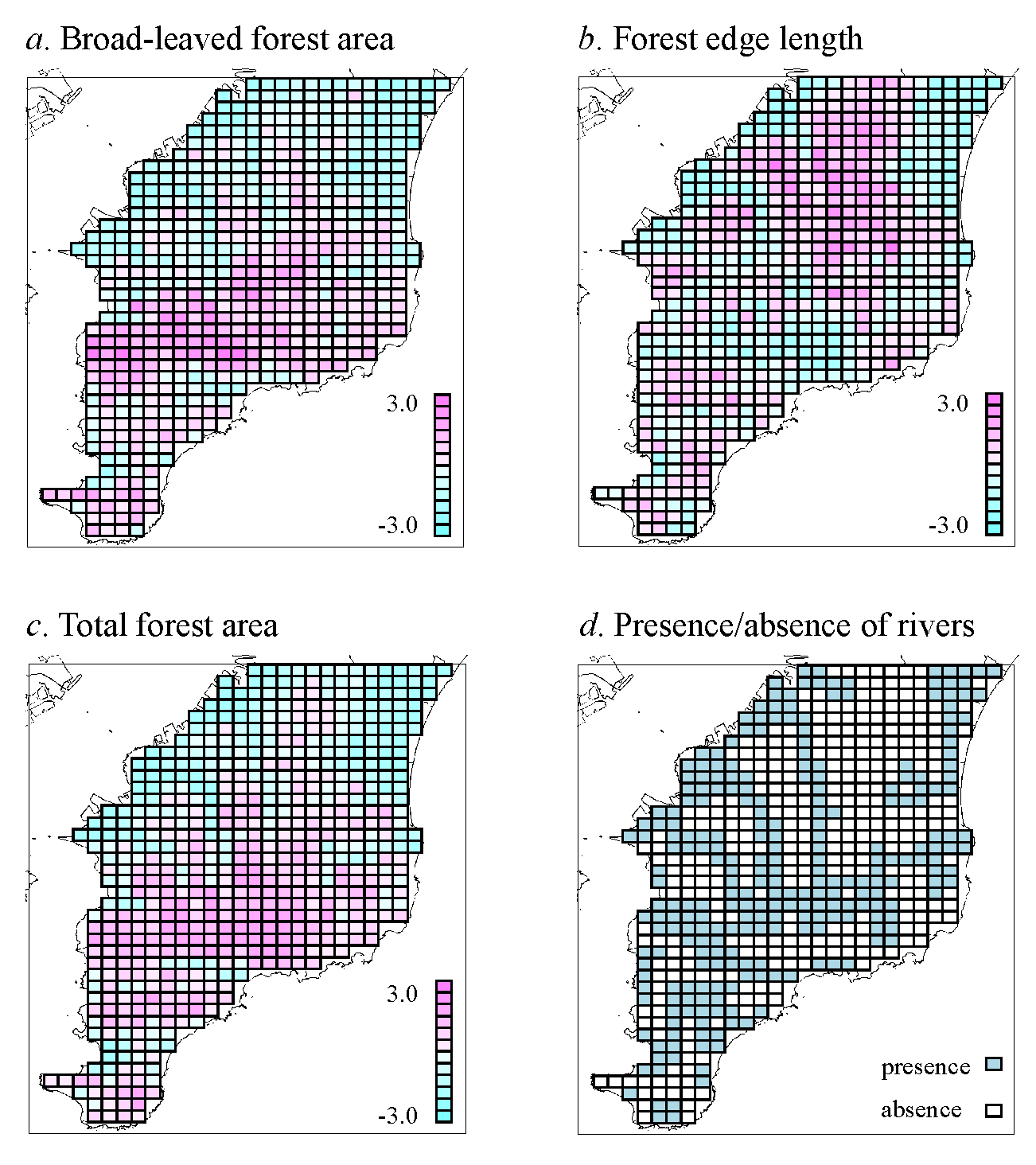


**Figure S4**. The environmental factors in south Boso peninsula. The panels represent standardized broad-leaf forest area (*a*), standardized forest edge length (*b*), standardized total forest area (i.e., both broad-leaf and conifer forest area) (*c*), and presence/absence of rivers (*d*). These environmental factors were extracted using geographical information systems.

**Appendix B: Specification of posterior and prior probability in our model**

**B-1. The specification of posterior probability**

In the appendix B, we describe the posterior probability of our model. For simplicity, we use two general functions, gamma function and delta function. These functions are defined as:

$$\Gamma\left( z \right)=\int_{0}^{\infty} dx x^{z-1}e^{-x},$$

$$\delta\left( x \right)=\left\{ \begin{aligned} 1 \left( x=0 \right) \\ 0 \left( x\neq0 \right) \end{aligned} \right.,$$

respectively. Then, the posterior probability of our model can be written out in full as follows (See the main text and Fig. 2 for the meanings of all parameters):

$$p\left( \gamma,\theta,\boldsymbol{\alpha},\boldsymbol{\beta} | \boldsymbol{F},\boldsymbol{B,}\boldsymbol{H}^{u}\boldsymbol{,P,A,}\boldsymbol{A}^{B} \right)$$

$$\propto\pi\left( \gamma\right) \pi\left( \theta\right) \pi\left( \boldsymbol{\alpha} \right) \pi\left( \boldsymbol{\beta} \right) p\left( \boldsymbol{F},\boldsymbol{B} | \gamma,\theta,\boldsymbol{\alpha},\boldsymbol{\beta,}\boldsymbol{H}^{u}\boldsymbol{,P,A,}\boldsymbol{A}^{B} \right)$$

$$=\pi\left( \gamma\right) \pi\left( \theta\right) \pi\left( \boldsymbol{\alpha} \right) \pi\left( \boldsymbol{\beta} \right) \prod_{t=2000}^{2010} p\left( \boldsymbol{F}_{t} | \gamma,\theta,{\boldsymbol{N}^{r}}_{t}\boldsymbol{,A} \right) p\left( \boldsymbol{B}_{t} | {\boldsymbol{N}^{r}}_{t}\boldsymbol{,A,}\boldsymbol{A}^{B} \right) p\left( {\boldsymbol{N}^{r}}_{t} | \boldsymbol{\alpha},\boldsymbol{\beta},{\boldsymbol{H}^{u}}_{t},\boldsymbol{P} \right)$$

$$=\pi\left( \gamma\right) \pi\left( \theta\right) \pi\left( \boldsymbol{\alpha} \right) \pi\left( \boldsymbol{\beta} \right) \pi\left( {\boldsymbol{N}^{r}}_{2000} \right)\prod_{t=2000}^{2010} p\left( \boldsymbol{F}_{t} | \gamma,\theta,{\boldsymbol{N}^{r}}_{t}\boldsymbol{,A} \right)p\left( \boldsymbol{B}_{t} | {\boldsymbol{N}^{r}}_{t}\boldsymbol{,A,}\boldsymbol{A}^{B} \right)\times$$

$$p\left( {\boldsymbol{N}^{r}}_{2000} | {\boldsymbol{N}^{r}}_{2000}\boldsymbol{,P,A} \right)\prod_{t=2000}^{2009} p\left( {\boldsymbol{N}^{r}}_{t+1} | \boldsymbol{\beta},{\boldsymbol{N}^{d}}_{t} \right) p\left( {\boldsymbol{N}^{d}}_{t} | {\boldsymbol{N}^{h}}_{t},{\boldsymbol{H}^{u}}_{t} \right) p\left( {\boldsymbol{N}^{h}}_{t} | \boldsymbol{\alpha}, {\boldsymbol{N}^{r}}_{t} \right)\times$$

$$=\pi\left( \gamma\right) \pi\left( \theta\right) \pi\left( \boldsymbol{\alpha} \right) \pi\left( \boldsymbol{\beta} \right) \pi\left( {\boldsymbol{N}^{\mathbf{d}}}_{2000} \right)\prod_{t=2000}^{2010} p\left( \boldsymbol{F}_{t} | \gamma,\theta,{\boldsymbol{N}^{r}}_{t}\boldsymbol{,A} \right)p\left( \boldsymbol{B}_{t} | {\boldsymbol{N}^{r}}_{t}\boldsymbol{,A,}\boldsymbol{A}^{B} \right)\times$$

$$p\left( {\boldsymbol{N}^{r}}_{2000} | {\boldsymbol{N}^{r}}_{2000}\boldsymbol{,P,A} \right)\prod_{t=2010}^{2009} p\left( {\boldsymbol{N}^{r}}_{t+1} | \boldsymbol{M},{\boldsymbol{N}^{d}}_{t} \right) p\left( \boldsymbol{H}_{t} | {\boldsymbol{N}^{h}}_{t},{\boldsymbol{H}^{\mathbf{u}}}_{t} \right) p\left( {\boldsymbol{N}^{h}}_{t} | \boldsymbol{r}, {\boldsymbol{N}^{r}}_{t} \right).$$

In this equation,

$$p\left( {\boldsymbol{N}^{h}}_{t} | \boldsymbol{r}, {\boldsymbol{N}^{r}}_{t} \right)=\prod_{i=1}^{578} \frac{\left( r_{i} {N^{r}}_{i,t} \right)^{{N^{h}}_{i,t}} e^{-r_{i} {N^{r}}_{i,t}}}{\Gamma\left( {N^{h}}_{i,t}+1 \right)},$$

$$p\left( {\boldsymbol{N}^{r}}_{t+1} | \boldsymbol{M},{\boldsymbol{N}^{d}}_{t} \right)=\sum_{{\boldsymbol{N}^{\mathbf{m}}}_{t}\boldsymbol{\geq0}} p\left( {\boldsymbol{N}^{\mathbf{d}}}_{t+1} | {\boldsymbol{N}^{\mathbf{m}}}_{t} \right) p\left( {\boldsymbol{N}^{\mathbf{m}}}_{t} | \boldsymbol{M},{\boldsymbol{N}^{\mathbf{h}}}_{t} \right)$$

$$=\sum_{{\boldsymbol{N}^{m}}_{t}\boldsymbol{\geq0}} \left\{ \prod_{j=1}^{578} \delta\left( {N^{r}}_{j,t+1}-\sum_{i=1}^{578} {N^{m}}_{ij,t} \right) \right\}\left\{ \prod_{i=1}^{578} \Gamma\left( {N^{d}}_{i,t}+1 \right)\prod_{j=1}^{578} \frac{{M_{i,j}}^{{N^{m}}_{ij,t}}}{\Gamma\left( {N^{m}}_{ij,t}+1 \right)} \right\},$$

$$p\left( {\boldsymbol{N}^{r}}_{2000} | {\boldsymbol{N}^{r}}_{2000}\boldsymbol{,P,A} \right)$$

$$=\prod_{i=1}^{578} \left( {1-P}_{i} \right)\delta\left( {N^{r}}_{j,2000} \right)+P_{i}\frac{\left( A_{i}\frac{\sum_{j\in n_{i}} {N^{r}}_{j,2000}}{\sum_{j\in n_{i}} A_{j}} \right)^{{N^{r}}_{i,2000}} e^{-A_{i}\frac{\sum_{j\in n_{i}} {N^{r}}_{j,2000}}{\sum_{j\in n_{i}} A_{j}}}}{\Gamma\left( {N^{r}}_{i,2000}+1 \right)},$$

$$p\left( \boldsymbol{F}_{t} | \gamma,\theta,{\boldsymbol{N}^{r}}_{t},A \right)=\prod_{i\in f_{t}} \frac{\Gamma\left( F_{i,t}+\theta\right)}{\Gamma\left( F_{i,t}+1 \right) \Gamma\left( \theta\right)}\left( \frac{\theta A_{i}}{\gamma{N^{r}}_{i,t}+\theta A_{i}} \right)^{\theta}\left( \frac{\gamma{N^{r}}_{i,t}}{\gamma{N^{r}}_{i,t}+\theta A_{i}} \right)^{F_{i,t}},$$

$$p\left( \boldsymbol{B}_{t} | {\boldsymbol{N}^{r}}_{t}\boldsymbol{,A,}\boldsymbol{A}^{\boldsymbol{B}} \right)=\prod_{i\in b_{t}} \frac{\left( {A^{B}}_{i}\frac{{N^{r}}_{i,t}}{A_{i}} \right)^{B_{i,t}} e^{-{A^{B}}_{i}\frac{{N^{r}}_{i,t}}{A_{i}}}}{\Gamma\left( B_{i,t}+1 \right)},$$

$$p\left( \boldsymbol{H}_{t} | {\boldsymbol{N}^{h}}_{t},{\boldsymbol{H}^{u}}_{t} \right)\boldsymbol{=}\prod_{k=1}^{66} \Gamma\left( {H^{u}}_{k,t}+1 \right)\prod_{i\in m_{k}} {\frac{1}{\Gamma\left( H_{i,t}+1 \right)}\left( \frac{{N^{h}}_{i,t}}{\sum_{i\in m_{u}} {N^{h}}_{i,t}} \right)}^{H_{i,t}}.$$

The prior probabilities, $\pi\left( \right)$, are described in the next section. Note that $p\left( {\boldsymbol{N}^{r}}_{t+1} | \boldsymbol{M},{\boldsymbol{N}^{d}}_{t} \right)$ cannot be calculated in realistic time using ordinary Markov chain Monte Carlo algorithm (MCMC). This is our reason for implementing Bayesian inference via the MCMC with particle filters.

**B-2. The specification of prior probability and MCMC initial values**

The prior probabilities were set using uniform or vague normal distributions (Table S1). As MCMC initial values of deer abundance in 2000, we used published estimates calculated from fecal pellet counts spatially interpolated by an inverse distance weighting method (Miyashita et al. 2008). The initial values of the other parameters were set randomly from their prior probability. We confirmed the weak influence of the prior distribution and the initial value on the posterior distribution by trace plot of each parameter (Fig. S5).

**References**

Miyashita T, Suzuki M, Ando D, Fujita G, Ochiai K, Asada M. 2008 Forest edge creates small-scale variation in reproductive rate of sika deer. *Popul. Ecol.* **50**, 111-120.

**Table S1.** The specification of prior probabilities.

|  | Prior probability | Other assumption |
| --- | --- | --- |
| ${N^{r}}_{i,2000}$ | U (0, 240) |  |
| $\gamma$ | U (0, 100) |  |
| $\theta$ | U (0, 100) |  |
| $\alpha_{0}$ | N (0, 100^2^) |  |
| $\alpha_{1}$ | N (0, 100^2^) |  |
| $\alpha_{2}$ | N (0, 100^2^) |  |
| $\beta_{0}$ | N (0, 100^2^) | $\beta_{0}<0$ |
| $\beta_{1}$ | N (0, 100^2^) |  |
| $\beta_{2}$ | N (0, 100^2^) |  |


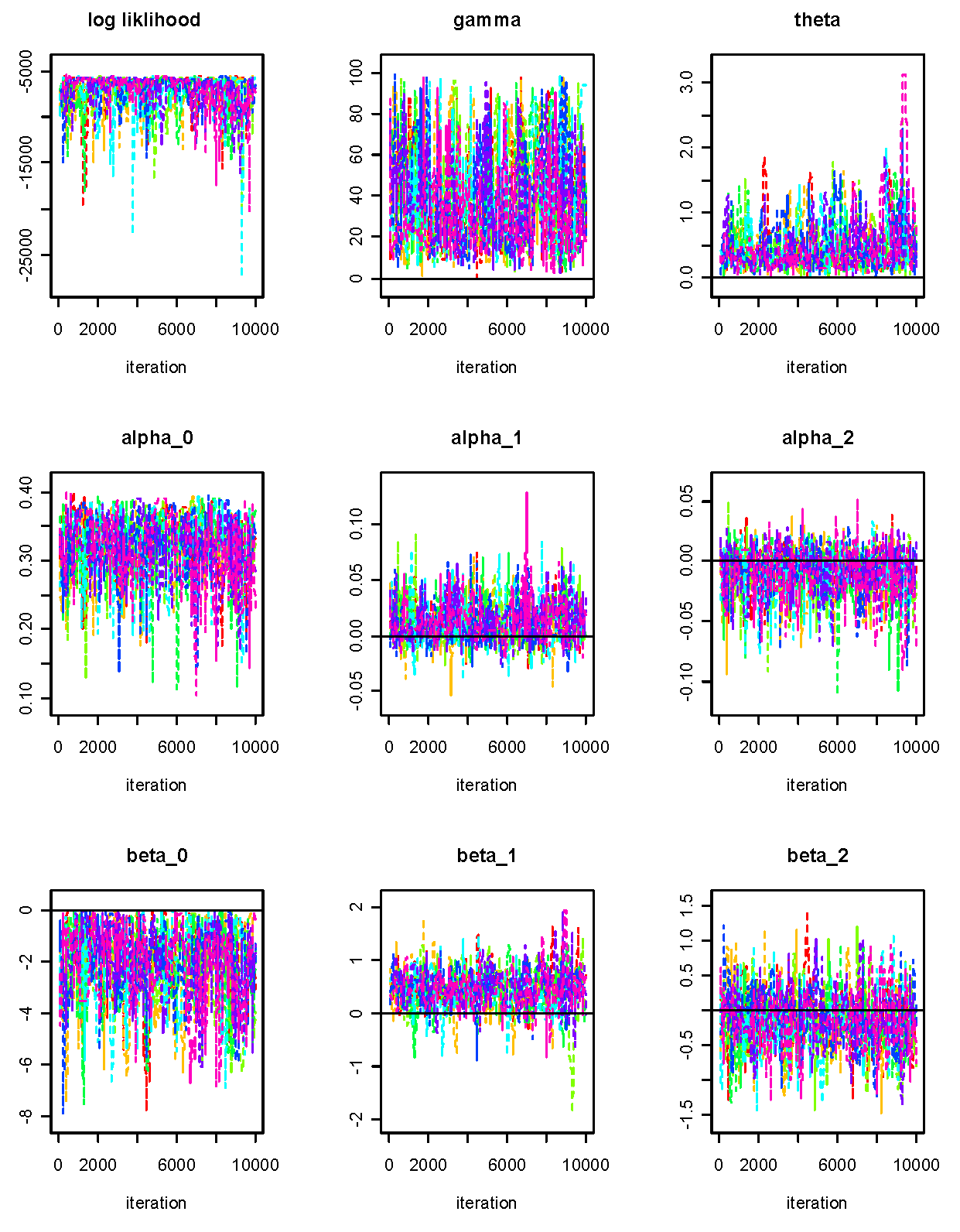


**Figure S5**. Trace plot of our MCMC simulations. Each panel represents trace plot of logarithm of likelihood or demographic parameter (see the detail of our demographic parameters in main text).

**Appendix C: Derivation and simulation experiments of modified approximation in PFMCMC algorithm**

**C-1. Ordinary and modified PFMCMC algorithms**

We used the Markov chain Monte Carlo algorithm with particle filters (PFMCMC; Andrieu et al. 2010; Knap & de Valpine 2012) to specify the complex spatiotemporal population dynamics model of sika deer in the south Boso peninsula. In this appendix, we describe the details of the PFMCMC algorithm and its modification. Let $\boldsymbol{x}_{t}$, $\boldsymbol{y}_{t}$, and $\boldsymbol{\theta}$ denote $t$-year state variables (e.g., estimated deer abundance), $t$-year data (e.g., observed deer abundance), and the other variables (e.g., demographic and observation parameters), respectively. The pseudocode of the ordinary procedure for PFMCMC algorithm is described in Algorithm 1 and Algorithm 2.

| **Algorithm 1: MCMC with particle filters** |
| --- |
| **given**:  data, $Y\boldsymbol{=}\left\{ \boldsymbol{y}_{1}\boldsymbol{,\ldots,}\boldsymbol{y}_{T} \right\}$  process model, $p\left( \boldsymbol{x}_{t} \vert\boldsymbol{x}_{t-1},\boldsymbol{\theta} \right)$  observation model, $p\left( \boldsymbol{y}_{t} \vert\boldsymbol{x}_{t},\boldsymbol{\theta} \right)$  prior probability, $\pi_{\boldsymbol{\theta}}\left( \boldsymbol{\theta} \right)$ |
| **begin** |
| sample initial values, $\boldsymbol{\theta}_{0}$**,** from an appropriate probability distributions |
| **for** $k=1$ **to** $K$ **do** (MCMC iterations) |
| sample state variables, $\boldsymbol{x}_{1,k,i}^{f}$, and compute $p\left( \boldsymbol{y}_{1} \vert\boldsymbol{\theta}_{k-1} \right)$ by Algorithm 2 |
| **for** $t=2$ **to** $T$ **do** (Time period) |
| predict state variables at $t$, $\boldsymbol{x}_{t,k,i}^{p} \sim p\left( \cdot\vert\boldsymbol{x}_{t-1,k,i}^{f},\boldsymbol{\theta}_{k-1} \right)$, for $i=1,2,\ldots,N$ |
| compute $w_{t,k,i}=p\left( \boldsymbol{y}_{t} \vert\boldsymbol{x}_{t,k,i}^{p},\boldsymbol{\theta}_{k-1} \right)$ |
| resample $\boldsymbol{x}_{t,k,i}^{f}$, from $\boldsymbol{x}_{t,k,i}^{p}$ according to $w_{t,k,i} / \sum_{i=1}^{N} w_{t,k,i}$ with replacement |
| compute $p\left( \boldsymbol{y}_{t} \vert\boldsymbol{y}_{1:t-1},\boldsymbol{\theta}_{k-1} \right)\approx{\sum_{i=1}^{N} w_{t,k,i}}/N$ |
| **end** |
| compute $p\left( \boldsymbol{y}_{1:T} \vert\boldsymbol{\theta}_{k-1} \right)= p\left( \boldsymbol{y}_{1} \vert\boldsymbol{\theta}_{k-1} \right)\prod_{t=2}^{T} p\left( \boldsymbol{y}_{t} \vert\boldsymbol{y}_{1:t-1},\boldsymbol{\theta}_{k-1} \right)$ |
| sample candidate values, $\boldsymbol{\theta}^{\boldsymbol{*}} \sim q\left( \cdot\vert\boldsymbol{\theta}_{k-1} \right)$ |
| compute $p\left( \boldsymbol{y}_{1:T} \vert\boldsymbol{\theta}^{\boldsymbol{*}} \right)$ substituting $\boldsymbol{\theta}^{\boldsymbol{*}}$ for $\boldsymbol{\theta}_{k-1}$ |
| compute $u=\min\left( 1,\frac{p\left( \boldsymbol{y}_{1:T} \vert\boldsymbol{\theta}^{\boldsymbol{*}} \right) \pi_{\boldsymbol{\theta}}\left( \boldsymbol{\theta}^{\boldsymbol{*}} \right)}{p\left( \boldsymbol{y}_{1:T} \vert\boldsymbol{\theta}_{k-1} \right) \pi_{\boldsymbol{\theta}}\left( \boldsymbol{\theta}_{k-1} \right)}\frac{q\left( \boldsymbol{\theta}_{k-1} \vert\boldsymbol{\theta}^{\boldsymbol{*}} \right)}{q\left( \boldsymbol{\theta}^{\boldsymbol{*}} \vert\boldsymbol{\theta}_{k-1} \right)} \right)$ |
| set $\boldsymbol{\theta}_{k}=\boldsymbol{\theta}^{\boldsymbol{*}}$ with probability $u$, otherwise $\boldsymbol{\theta}_{k}=\boldsymbol{\theta}_{k-1}$ |
| **end** |
| **End** |
| **return:** posterior distributions, $\boldsymbol{\Theta=}\left\{ \boldsymbol{\theta}_{1},\ldots,\boldsymbol{\theta}_{K} \right\}$ |

| **Algorithm 2: Ordinary sampling of state variables at** $\boldsymbol{t=1}$ |
| --- |
| **given**:  data at $t=1$, $\boldsymbol{y}_{1}$  parameter, $\boldsymbol{\theta}$ ($\mathbf{=}\boldsymbol{\theta}_{k-1}$ in Algorithm 1)  observation model, $p\left( \boldsymbol{y}_{t} \vert\boldsymbol{x}_{t},\boldsymbol{\theta} \right)$  prior probability, $\pi_{\boldsymbol{x}}\left( \boldsymbol{x}_{1} \vert\boldsymbol{\theta} \right)$ |
| **begin** |
| sample state variables, $\boldsymbol{x}_{1,i}^{p} \sim\pi_{x}\left( \cdot\vert\boldsymbol{\theta} \right)$ for $i=1,2,\ldots,N$ |
| compute $w_{i}=p\left( \boldsymbol{y}_{1} \vert\boldsymbol{x}_{1,i}^{p},\boldsymbol{\theta} \right)$ |
| resample $\boldsymbol{x}_{1,i}^{f}$, from $\boldsymbol{x}_{1,i}^{p}$ according to $w_{i} / \sum_{i=1}^{N} w_{i}$ with replacement |
| compute $p\left( \boldsymbol{y}_{1} \vert\boldsymbol{\theta} \right)\approx{\sum_{i=1}^{N} w_{i}}/N$ |
| **End** |
| **return:** $\boldsymbol{x}_{1,i}^{f}$ and $p\left( \boldsymbol{y}_{1} \vert\boldsymbol{\theta} \right)$ ($=\boldsymbol{x}_{1,k,i}^{f}$, and $p\left( \boldsymbol{y}_{1} \vert\boldsymbol{\theta}_{k-1} \right)$ in Algorithm 1) |

If the dimension of the state variables is relatively low, the ordinary procedure performs well to obtain the posterior probability, $p\left( \boldsymbol{\theta} | \boldsymbol{y}_{1:T} \right)$. However, the performance of this procedure becomes worse as the dimensionality of the state variables increases because only a few $w_{i}$ become large and the variation in $\boldsymbol{x}_{1,i}^{f}$ rapidly shrinks (i.e., particle shrinkage) in Algorithm 2. To prevent the particle shrinkage, we modified ordinary PFMCMC procedure as the pseudocode of Algorithm 3. The modified procedure is validated because we can derive as follows:

$$p\left( \boldsymbol{y}_{1} | \boldsymbol{\theta} \right)={p\left( \boldsymbol{y}_{1} | \boldsymbol{\theta} \right)}/{\int d\boldsymbol{x}_{1} p\left( \boldsymbol{x}_{1} | \boldsymbol{\theta} \right)}=1/{\int d\boldsymbol{x}_{1} \frac{p\left( \boldsymbol{x}_{1} | \boldsymbol{y}_{1},\boldsymbol{\theta} \right)}{p\left( \boldsymbol{y}_{1} | \boldsymbol{x}_{1},\boldsymbol{\theta} \right)}}$$

$$=\frac{1}{E_{\boldsymbol{x}_{1}|\boldsymbol{y}_{1},\boldsymbol{\theta}}\left[ 1/{p\left( \boldsymbol{y}_{1} | \boldsymbol{x}_{1},\boldsymbol{\theta} \right)} \right]}\approx N/{\sum_{i=1}^{N} \frac{1}{p\left( \boldsymbol{y}_{1} | \boldsymbol{x}_{1,i}^{f},\boldsymbol{\theta} \right)}}.$$

Because we sample $\boldsymbol{x}_{i}^{f}$ from $p\left( \cdot| \boldsymbol{y}_{1}\boldsymbol{,}\boldsymbol{\theta}_{k-1} \right)$ directly, the variation in particles never shrinks at year $t=1$ in Algorithm 3.

| **Algorithm 3: Modified sampling of state variables at** $\boldsymbol{t=1}$ |
| --- |
| **given**:  data at $t=1$, $\boldsymbol{y}_{1}$  parameter, $\boldsymbol{\theta}$ ($\mathbf{=}\boldsymbol{\theta}_{k-1}$ in Algorithm 1)  observation model, $p\left( \boldsymbol{y}_{t} \vert\boldsymbol{x}_{t},\boldsymbol{\theta} \right)$  prior probability, $\pi_{\boldsymbol{x}}\left( \boldsymbol{x}_{1} \vert\boldsymbol{\theta} \right)$ |
| **begin** |
| compute $p\left( \boldsymbol{x}_{1} \vert\boldsymbol{y}_{1}\boldsymbol{,}\boldsymbol{\theta}_{k-1} \right)$ from $p\left( \boldsymbol{y}_{1} \vert\boldsymbol{x}_{1},\boldsymbol{\theta} \right)$ and $\pi_{\boldsymbol{x}}\left( \boldsymbol{x}_{1} \vert\boldsymbol{\theta} \right)$ by Bayes’ theorem |
| sample state variables, $\boldsymbol{x}_{1,i}^{f} \sim p\left( \cdot\vert\boldsymbol{y}_{1}\boldsymbol{,}\boldsymbol{\theta}_{k-1} \right)$ for $i=1,2,\ldots,N$ |
| compute $p\left( \boldsymbol{y}_{1} \vert\boldsymbol{\theta} \right)\approx N/\left\{ \sum_{i=1}^{N} 1/{p\left( \boldsymbol{y}_{1} \vert\boldsymbol{x}_{1,i}^{f}\boldsymbol{,}\boldsymbol{\theta}_{k-1} \right)} \right\}$ |
| **end** |
| **return:** $\boldsymbol{x}_{1,i}^{f}$ and $p\left( \boldsymbol{y}_{1} \vert\boldsymbol{\theta} \right)$ ($=\boldsymbol{x}_{1,k,i}^{f}$, and $p\left( \boldsymbol{y}_{1} \vert\boldsymbol{\theta}_{k-1} \right)$ in Algorithm 1) |

**C-2. The performance of the modified procedure**

We compared the performances of the ordinary and modified procedure (i.e., Algorithm 2 and Algorithm 3) using a simple toy example. Let $x$ and $y$ be the state vector and data vector, respectively. In this example, we set prior probability $\pi\left( x \right)$ and data model $p\left( y | x \right)$ to normal distribution:

$$\pi\left( x \right) : N \left( x | 0,{100}^{2} \right),$$

$$p\left( y | x \right) : N \left( 0 | x,{10}^{2} \right).$$

Because of conjugacy, the filtered probability is expressed as

$$p\left( x | y \right)=\frac{p\left( y | x \right) \pi\left( x \right)}{\int dx p\left( y | x \right) \pi\left( x \right)} : N\left( x | 0,\frac{{1000}^{2}}{{10}^{2}+{100}^{2}} \right).$$

Our objective is to approximate the marginalized probability:

$$p\left( y \right)=\int dx p\left( y | x \right) \pi\left( x \right).$$

The performance of both approximations are represented in Fig. S6-7. When the dimension of the state variables is 1, the ordinary algorithm outperforms the modified algorithm (Fig. S6). However, as the dimension of the state vector increases, the modified algorithm increasingly outperforms the ordinary algorithm (left panel in Fig. S7). As shown in Fig. S6, the modified algorithm always has positive bias for finite number of particle, while the ordinary algorithm always has negative bias. It is important to note that the modified algorithm can prevent particle shrinkage by directly sampling particles from the filtered distribution (right panel in Fig. S7). Thus, our modified algorithm works so much more effectively than the ordinary algorithm when implementing particle filter with high dimensions of state variables. The computational efficiency of the modified algorithm largely depends on computation of filtered probability. But our experience is that computational load (elapsed time and memory) of additional particles for ordinary algorithm is often higher than that of filtered probability calculation. In this section, we provide only simple numerical experiment. Future works are needed to evaluate performance of our algorithm for more complex numerical experiments with realistic conditions.

**Reference**

Andrieu C, Doucet A, Holenstein R. 2010 Particle Markov chain Monte Carlo methods. *J. R. Statist. Soc. B* **72**, 269-342.

Knape J, de Valpine P. 2012 Fitting complex population models by combining particle filters with Markov chain Monte Carlo. *Ecology* **93**, 256-263.


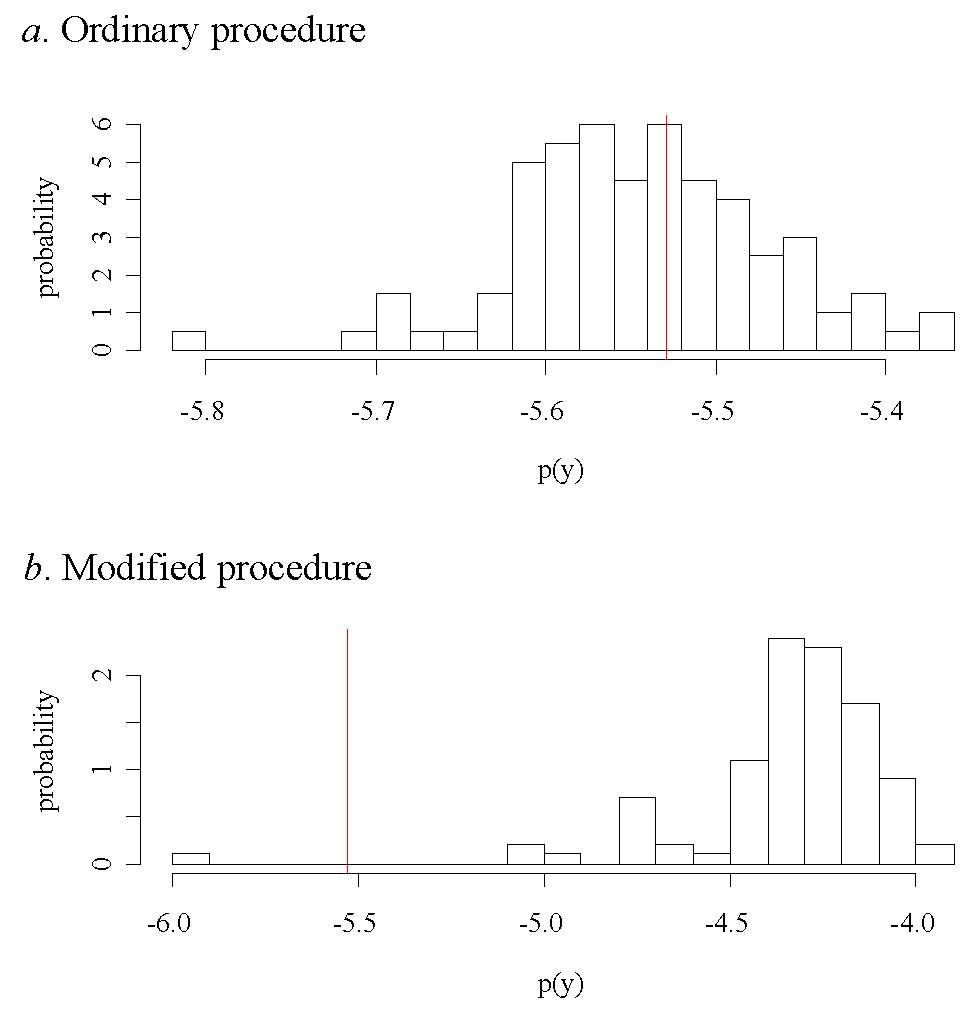


**Figure S6.** The performance of the ordinary (*a*) and modified (*b*) procedure when the dimension of state vector is 1. These histograms represent $p\left( y \right)$ calculated from ordinary and modified procedures (1000 replicates). The red vertical line represents the theoretical value.


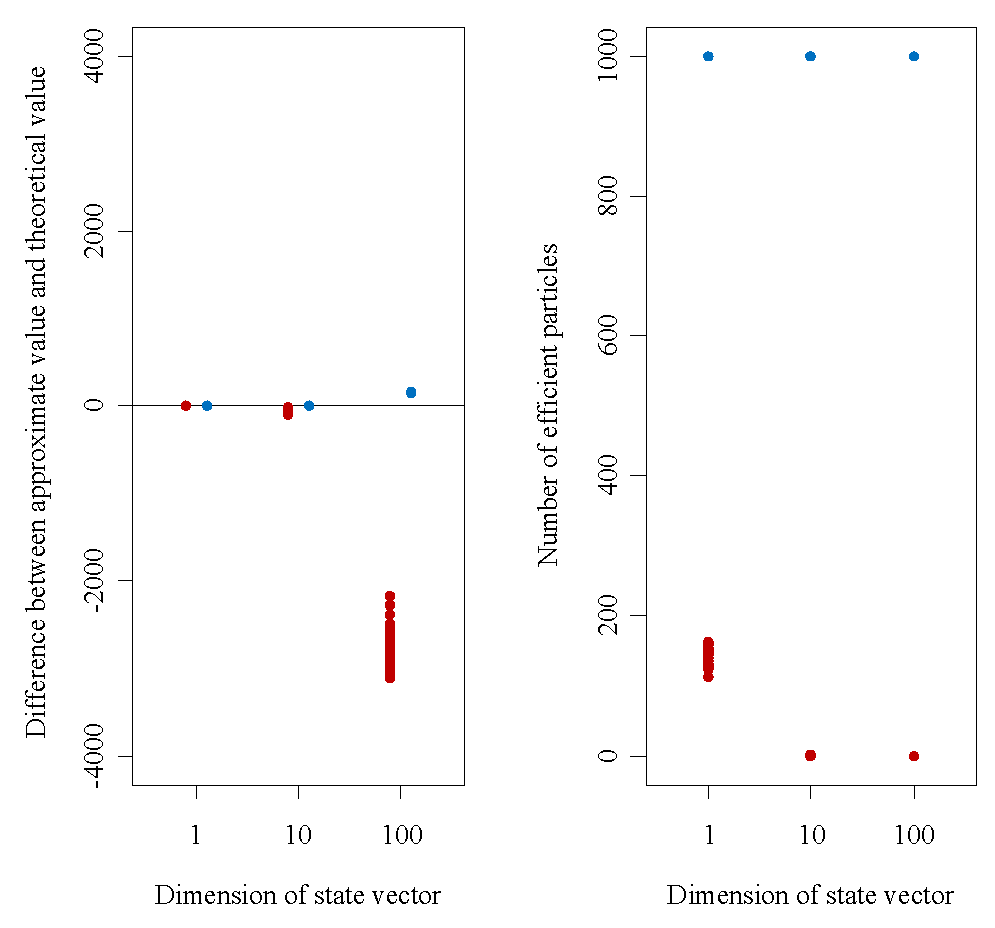


**Figure S7.** The performance of the ordinary and modified procedure with increasing dimension of state vector. The red and blue circles represent approximation of ordinary and modified procedures, respectively (1000 replicates).
